# Inhibition of Vicariously Learned Fear in Children Using Positive Modeling and Prior Exposure

**DOI:** 10.1037/abn0000131

**Published:** 2015-12-14

**Authors:** Chris Askew, Gemma Reynolds, Sarah Fielding-Smith, Andy P. Field

**Affiliations:** 1 Department of Psychology, Kingston University; 2 School of Psychology, University of Sussex

**Keywords:** anxiety, vicarious learning, childhood fears, fear prevention, observational learning

## Abstract

One of the challenges to conditioning models of fear acquisition is to explain how different individuals can experience similar learning events and only some of them subsequently develop fear. Understanding factors moderating the impact of learning events on fear acquisition is key to understanding the etiology and prevention of fear in childhood. This study investigates these moderators in the context of vicarious (observational) learning. Two experiments tested predictions that the acquisition or inhibition of fear via vicarious learning is driven by associative learning mechanisms similar to direct conditioning. In Experiment 1, 3 groups of children aged 7 to 9 years received 1 of 3 inhibitive information interventions—psychoeducation, factual information, or no information (control)—prior to taking part in a vicarious fear learning procedure. In Experiment 2, 3 groups of children aged 7 to 10 years received 1 of 3 observational learning interventions—positive modeling (immunization), observational familiarity (latent inhibition), or no prevention (control)—before vicarious fear learning. Results indicated that observationally delivered manipulations inhibited vicarious fear learning, while preventions presented via written information did not. These findings confirm that vicarious learning shares some of the characteristics of direct conditioning and can explain why not all individuals will develop fear following a vicarious learning event. They also suggest that the modality of inhibitive learning is important and should match the fear learning pathway for increased chances of inhibition. Finally, the results demonstrate that positive modeling is likely to be a particularly effective method for preventing fear-related observational learning in children.

## Abstract (general public)

The studies demonstrate that positive modeling and prior exposure to a stimulus are effective methods to prevent vicarious fear learning in 7 to 10-year-olds. This confirms that positive modeling is a useful part of prevention programs and that associative learning mechanisms underpin its effectiveness. The findings also explain why not everyone develops fear following a negative vicarious learning event.

Anxiety disorders are common; they have a prevalence of over 25% in the United States, making them the most frequent psychological disorder in adulthood (Coles & Coleman, 2010; Kessler et al., 2005) and adolescence (Copeland, Angold, Shanahan, & Costello, 2014; Essau, Conradt, & Petermann, 2000; Merikangas et al., 2010). Most anxiety disorders begin during childhood, and of these, specific phobias begin particularly early (Kessler et al., 2005). Animal fears are among the earliest phobias to develop (Öst & Treffers, 2001), with 62% beginning between the ages of 5 to 9 years (Öst, 1987). Although anxiety disorders often start relatively early in life, individuals do not typically seek treatment until much later: Only 13.7–28.0% of Europeans seek treatment in the first year of onset, and numbers are even lower in the United States, New Zealand, and Japan (Wang et al., 2007). The delay for seeking treatment for anxiety disorders after onset is lengthy; typical median delays are around 10–28 years in Europe, 23 years in the United States, and can be even higher elsewhere (Wang et al., 2005). Reasons for delay are likely to vary, but include lack of recognition of symptoms and knowledge about mental illness and treatment (i.e., low mental health literacy; Jorm, 2000, 2012). In particular, early-onset anxiety disorders, like phobia, are associated with some of the longest delays before treatment (Wang et al., 2005), which may be the result of lower mental health literacy during early stages of life, and possibly also the ability of individuals with phobias to avoid their fear-provoking stimulus. Consequently, individuals often suffer the negative effects of fear and phobia for many years. It has therefore been recommended that future interventions should be aimed early, at children and adolescents (Kessler et al., 2005). Focusing interventions even earlier, at preventing fear before it has developed rather than treating fear, may be even more effective. Critical to this aim is research with nonclinical, typically developing children to determine the mechanisms driving how and when fears do, or do not, develop.

The importance of prevention of psychological disorders is now widely recognized, as is the need for interventions to be evidence-based (World Health Organization, WHO, 2013). While many psychological prevention programs have been shown to be effective for preventing mental disorders (see, e.g., Saxena, Jané-Llopis, & Hosman, 2006; WHO, 2004), they are not typically theory-based at the level of mechanisms underpinning effects. That is, studies often convincingly demonstrate that the intervention programs work as a whole but the specific underlying mechanisms of individual prevention methods are not always clearly defined or explored. Preventative interventions typically combine a range of techniques including, for example, psychoeducation and modeling (Barrett, Cooper & Teoh, 2014). However, the effectiveness of the individual constituents, and the mechanisms through which therapeutic change occurs, are often not examined. For a specific facet of an anxiety prevention program to be deemed effective it is important to demonstrate that it had a causal contribution in reducing or protecting against anxiety. It is also useful to demonstrate how it had the effect (i.e., the psychological mechanism of change). This study aims to address this need by systematically testing theoretical predictions about vicarious learning that are relevant to the prevention of fears.

There is good evidence that vicarious learning experiences, in which someone responding negatively to a stimulus or situation is observed, can lead to the development of fear and phobias in children (see Askew & Field, 2008, for a review). Experimental studies have demonstrated that vicarious fear learning can lead to increases in children’s fear beliefs (e.g., Askew, Dunne, Özdil, Reynolds, & Field, 2013 [aged 6–11 years]; Askew & Field, 2007 [aged 7–9 years]; Dunne & Askew, 2013 [aged 6–10 years]), avoidance behavior (e.g., Askew & Field, 2007; Askew et al., 2013 [aged 6–11 years]; Dubi, Rapee, Emerton, & Schniering, 2008 [aged 15–20 months]; Dunne & Askew, 2013; Egliston & Rapee, 2007 [aged 12–20 months]; Gerull & Rapee, 2002 [aged 15–20 months]), heart rate, and attentional bias (G. Reynolds, Field, & Askew, 2014, 2015b [aged 7–9 and 6–10 years]) for stimuli. Askew and Field (2007) developed a vicarious learning paradigm that involves showing children pictures of novel animals together with pictures of fearful faces. The procedure is a simple analogue of how fears are vicariously acquired in the real world and presents a safe model for investigating the mechanisms underpinning fear learning to determine when and why learning occurs and test early intervention and fear-prevention techniques. Findings from the paradigm have been explained using theories of associative learning: Vicarious learning is driven by a conditioned stimulus (CS) becoming associated with a model’s fearful response, which acts as a negative unconditioned stimulus (US, e.g., Askew & Field, 2007, 2008; Mineka & Cook, 1993; Olsson & Phelps, 2007). Thus, like conditioning, vicarious learning operates through CS–US associations.

One of the challenges for traditional conditioning models of fear and phobias was that not everyone develops a fear after a negative learning event with a stimulus. Models could not explain, for example, why not everyone who has painful dental treatment develops dental phobia (Lautch, 1971), and not everyone who has a painful experience with dogs later fears them (Di Nardo et al., 1988). Likewise, for vicariously learned fears, self-report evidence suggests that several individuals experiencing similar vicarious learning events will not all go on to develop fear. For example, Doogan and Thomas (1992) found no difference in the number of dog-fearful and nonfearful adults recalling fear-related vicarious learning events involving dogs, suggesting a vicarious learning experience is not always sufficient to cause fear to develop.

Contemporary conditioning models (e.g., Davey, 1997; Field, 2006; Field & Purkis, 2011; Mineka & Zinbarg, 2006) can explain this finding. One established feature of conditioning is the effect of prior learning history about the CS on acquisition of a conditioned response (CR; Field, 2006). The success of fear conditioning can be influenced by expectancies about the relationship of a CS to a US prior to learning (see, e.g., Davey, 1997; Field, 2006; Field & Purkis, 2011): The strength of a CS–US association is influenced by the degree to which an individual believes the US is predicted by the CS (Alloy & Tabachnik, 1984) and the strength of the CR is determined by the strength of this association (Rescorla, 1980). Therefore, any learning history influencing beliefs about the CS–US relationship will affect the strength of the CR acquired during learning (Field, 2006; Field & Purkis, 2011). For example, compared to a child that already believes dogs are potentially threatening (i.e., dogs are a CS that predicts a painful or frightening US), a child that has neutral beliefs about the outcome of an experience with dogs is likely to learn a weaker CS–US association with a less potent CR during a traumatic learning event involving a dog. Field and Purkis (2011) have argued that whether the learning event is direct, informational, or vicarious, the underlying associative learning mechanisms are identical. If this is the case, previous learning history with a stimulus should be important in vicarious learning as it is in direct conditioning. Thus children who receive nonthreatening information about a stimulus would be predicted to create neutral or positive learning expectancies, and these should protect them from developing a fear response during a vicarious fear learning event involving the stimulus.

The current studies test theoretical predictions of contemporary conditioning models in the context of vicarious learning. If previous nontraumatic learning history is also important in vicarious learning this would confirm that underlying mechanisms are shared with direct conditioning and explain why some children can experience a vicarious fear learning event but not develop fear (e.g., Doogan & Thomas, 1992). If theoretical predictions of conditioning models hold in a vicarious learning context, this would also allow testable predictions to be made about the prevention of vicarious learning, potentially having implications for the development of future interventions. Given that vicarious learning can sometimes cause childhood fears, and fears and phobias are known to begin at this time and remain untreated for many years, it would be beneficial to understand the factors determining why vicarious fear learning occurs and how it can be prevented.

One additional factor that may influence the effectiveness of vicarious fear learning is the modality, or “pathway”, via which a learning history has been acquired. An analogous example of this can be seen in postlearning fear-reversal interventions: Kelly, Barker, Field, Wilson, and S. Reynolds (2010) investigated unlearning of fear responses acquired via verbal information and found that fear reversal was more successful when the unlearning intervention was also verbal; that is, when the fear learning and fear reversal pathways matched. Similarly, it is possible that a positive learning history acquired via an observational learning pathway might be more successful at inhibiting vicarious fear learning than a verbally acquired learning history.

Therefore, two experiments investigating the inhibition of vicarious fear learning via two distinct pathways are described. In Experiment 1 children received one of two types of preventative information or no prevention, and in Experiment 2 children experienced one of two observational learning preventions or no prevention. Askew and Field’s (2007) procedure was used to test whether vicarious fear learning occurred. Based on current knowledge about CS–US associative learning, and the prediction that vicarious learning shares the same underlying mechanisms as direct conditioning, it was anticipated that the interventions would successfully prevent the acquisition of fear during vicarious learning. Moreover it was thought that neutral or positive information presented observationally might be more effective because of the shared pathway with the fear learning procedure.

## Experiment 1

One reason why some children do not develop fear responses during a fear-related vicarious learning event may be because they do not interpret the US (the model’s response to the stimulus) as intense enough for learning to occur; for example, if they do not believe that the scared model is a reliable source of information about how threatening the stimulus is. One way to test this would be to use information to manipulate children’s interpretation of models’ responses. Psychoeducation may be one method to reduce negative learning expectancies. It can be used to help children and their families understand how anxiety develops, how it is maintained, and how it is treated, as well as to teach children to recognize emotional and physiological signs of anxiety and develop coping strategies. Psychoeducation is one of the tools used by clinicians to prevent (Barrett et al., 2014) or treat childhood disorders by, for example, correcting threat-related myths and false assumptions about a feared stimulus, as well as catastrophic expectancies (Davis, Ollendick, & Öst, 2009). For example, it can successfully reduce spider fear in 8- to 10-year-olds (Leutgeb, Schaider, & Schienle, 2012). If psychoeducation could be used to reduce how threatening children interpret a model’s fearful responses to be (i.e., reduce the intensity of the US), associative learning models would predict a reduction in the learnt fear response to CSs that become associated with the US. This devaluation of the US could explain the resilience that some children show to fear-related vicarious learning and clarify the mechanisms underpinning psychoeducation as a prevention and treatment method. Thus in Experiment 1, children were divided into three groups and one group was given information about how sometimes individuals may respond to a stimulus with fear even though the stimulus is not actually threatening or dangerous. A specific example was given in which a child became afraid of a nondangerous stimulus after observing an adult acting afraid of it. Between the ages of 4 to 6 years children typically begin to develop an understanding that a person’s emotional responses to a situation are determined, at least in part, by their beliefs (Pons, Harris, & de Rosnay, 2004). Consequently, it was predicted that this information would lead children (aged 7 to 9 years) to devalue models’ fear responses (US) to stimuli (CSs) during vicarious learning, making the outcome less threatening and hence reduce fear learning (CR). A second group of children were given nonthreatening factual information about the CS to create neutral expectancies. A third (control) group was given irrelevant information, presented in a similar manner and format to the other two conditions, but that was not related to the CS or US. Given predictions from conditioning models, and that vicarious learning has been argued to share the same characteristics (Field & Purkis, 2011) it was expected that psychoeducation and nonthreatening information would inhibit vicarious fear learning compared to the control group.

### Method

#### Participants

Fifty-six (30 boys, 26 girls) children aged between 7.83 and 9.83 years (*M* = 8.63 years, *SD* = 0.53) were recruited from a primary school in West Sussex, U.K. Parents were not asked to provide sociodemographic information but school records showed that children attending this village school were predominantly of White British background and fluent English speakers. The number of socioeconomically disadvantaged children attending the school was well below the national average. The school and parents/caregivers gave informed opt-in consent for children to take part and children gave their verbal assent. Children were randomly allocated to three information groups. There were initially 61 children but one child did not complete the entire procedure and four others were excluded because they stated at the end of the experiment that they were familiar with the animals. Thus there were 20 children in the psychoeducation group, 17 in the factual information group, and 19 children in the control group. A one-way independent ANOVA (Brown-Forsythe) found no significant difference between trait anxiety scores in the three groups (psychoeducation: *M* = 36.45, *SD* = 8.24; factual information: *M* = 34.71, *SD* = 4.04; control: *M* = 33.53, *SD* = 3.85), *F*(2, 36.97) = 1.30, *p* = .28. There was also no significant difference in mean ages in the three groups (psychoeducation: *M* = 8.58 years, *SD* = 0.56; factual information: *M* = 8.65 years, *SD* = 0.49; control: *M* = 8.66 years, *SD* = 0.55), *F*(2, 53) = 0.11, *p* = .90.

#### Materials

##### Stimuli

###### Animals (CSs)

Pictures (400 × 400 pixels) of three Australia marsupials (the quoll, quokka, and cuscus) were used as novel animal stimuli. These stimuli have been successfully used in previous similar studies (e.g., Askew & Field, 2007; Dunne & Askew, 2013; G. Reynolds et al., 2014) and U.K. children in this age group are typically unfamiliar with them. Three pictures of each animal were used making a total of nine different images in all.

###### Faces (USs)

Emotional face stimuli consisted of 20 portrait photographs (400 × 513 pixels) taken from the NimStim Set of Facial Expressions (Tottenham et al., 2009). There were five female and five male faces displaying happy facial expressions, and five female and five male faces displaying fearful facial expressions.

##### Measures

###### State–Trait Anxiety Inventory for Children (STAIC)

The trait scale from the STAIC (Spielberger, Gorsuch, Lushene, Vagg, & Jacobs, 1973) was used to measure children’s trait anxiety (how the child “usually feels”). The scale consists of a 20 statements about how boys and girls sometimes feel (e.g., “I worry about making mistakes”) and children are asked to decide whether each statement is true for them: *hardly ever* = 1, *sometimes* = 2, or *often* = 3. The scale typically shows good internal consistency, with Cronbach’s alphas of .81 for females and .78 for males, and test–retest reliability coefficients of .71 for females and .65 for males reported by Spielberger et al. (1973).

###### Fear Beliefs Questionnaire (FBQ)

A computerized version of the 21-item version of the FBQ (Field & Lawson, 2003) was used and consisted of seven identical questions (four reverse-scored) for each of the three animals: quoll, quokka, and cuscus. Items are designed to measure children’s fear-related beliefs for the animals; for example, “Would you keep your distance if you saw a quoll/quokka/cuscus?” or “Would you be happy to have a quoll/quokka/cuscus for a pet?” (reverse-scored). Children responded on a 5-point Likert-type response scale (0 = *no, not at all*; 1 = *no, not really*; 2 = *do not know/neither*; 3 = *yes, probably*; 4 = *yes, definitely*). Scores were averaged across items creating a mean fear belief score for each animal ranging from 0–4, with higher scores indicating higher levels of fear beliefs. Internal consistencies (Cronbach’s alpha) were high before learning: α = .74 (Cuscus subscale), .79 (Quokka subscale) and .82 (Quoll subscale); and after learning, αs = .89, .90 and .83, respectively.

###### Nature Reserve Task (NRT)

An adaptation of Field and Storksen-Coulson’s (2007) NRT was used to measure children’s avoidance preferences for the animals. The NRT consisted of a circular wooden board (diameter = 37.5 cm) covered in green material that children were asked to imagine was a nature reserve where the three animals lived. A rectangular 2-D cardboard photograph of each animal (3.4 × 3.4 cm with a 1-cm base as a stand) was placed, one at a time, at a specific point on the circumference of the board. The order of presentation of each animal was counterbalanced across children. Children were asked to imagine they were visiting the nature reserve and place a male (for boys) or a female (for girls) Lego figure (1.5 × 3.5 × 0.8 cm) where they would most like to be. The distance (in mm) between the center of the figure and the center of the animal picture was measured for each of the three animals to indicate children’s approach or avoidance preferences for that animal.

##### Preventative information manipulation

###### Psychoeducation

A nine-page psychoeducational leaflet (A5 size) with nine color pictures of cartoon characters was created to communicate information regarding the unreliability of facial expressions as an indicator of actual threat, the highly subjective nature of fears, and how fear of something might be vicariously transmitted from one person to another even though it is not dangerous. This leaflet was based on the format used by “Coping Cat” (Kendall & Hedtke, 2006), a popular CBT-based fear and anxiety-prevention program for children age 7–13, and began with an adult character called Jack who is afraid of a lizard. A lizard, and not one of the animals seen during vicarious learning, was chosen because the object of the information in this condition was to devalue the US (the model’s response), but not the CS. In a series of text and pictures a child was shown acting fearful of a lizard after seeing Jack afraid. It was explained that sometimes fear is useful when things are dangerous “like fire or crocodiles,” but sometimes people might be afraid of something just because they saw someone else acting afraid and assume that it must be dangerous when it is not. That person might also, it was explained, be afraid because they saw someone else acting afraid and this process could go on from person to person forever. Children were advised at the end of the information leaflet to remember that sometimes we are afraid of things that are not dangerous and if they are not sure, it is better to find out if something really is dangerous by asking a parent or teacher rather than feel afraid unnecessarily.

###### Factual information

A similarly formatted nine-page leaflet (A5 size) with nine color pictures of quolls, cuscuses, and quokkas was created to communicate factual information about the marsupials. For the purpose of the information leaflet, fairly neutral and nonthreatening commonalities between the three animals such as size, nocturnal lifestyle, diet, and predators were emphasized. For example, “Cuscuses, Quolls and Quokkas are nocturnal, which means that they sleep during the day and search for food at night. They have very good eyesight to help them see in the dark” and “Cuscuses, Quolls and Quokkas are quite small—about half a meter long. They like to eat fruit, nuts, leaves, insects and smaller animals, but not people!”

###### Unrelated information (control)

A third nine-page leaflet (A5 size) with nine color pictures was created to communicate information about ancient Greek gods and goddesses. This was a topic entirely unrelated to the CS, US, or experimental hypothesis and so served as a control manipulation. The style was comparable to the other leaflets with color cartoon pictures and informative text; for example, “Ancient Greeks believed that gods and goddesses watched over them. The gods were like humans, but immortal (they lived forever) and much more powerful” and “Zeus was the king of the Greek gods, and ruled all of the heavens. He threw thunderbolts to punish anyone who disobeyed him.”

#### Vicarious learning

Vicarious learning consisted of a total of 30 animal-face pairing trials presented in random order. Each child saw 10 trials in which one of the animal pictures was presented together with scared face pictures (fear-paired trials), 10 trials in which one of the animals was seen with happy faces (happy-paired trials), and 10 trials of the third animal alone on the screen (unpaired control trials). The type of emotional face (fear, happy, or none) children saw with each particular animal (quoll, quokka, or cuscus) was determined by the counterbalancing group they were in. A single animal–face paring trial was 2-s long and began with a randomly chosen animal picture being presented alone on the screen for 1 s on a randomly determined side of the screen. The animal continued on the screen for a further 1 s while the emotional face was presented on the opposite of the screen. Unpaired animals trials were identical except that no face appeared and the animal remained on the screen alone for 2 s. Between each trial there was a randomly determined 2-s to 4-s intertrial interval (see, e.g., Askew & Field, 2007; Askew & Field, 2008).

#### Procedure

Children first completed the STAIC and the FBQ. The FBQ and later vicarious learning procedure were automated on a Dell Inspiron 1300 laptop computer with a 13-inch monitor running Windows XP, using software custom-written by the fourth author in VisualBasic.net with ExacTicks 1.10 (Ryle Design, 1997). Following the FBQ, children received one of three types of information depending on the group they were randomly allocated to: a) psychoeducation; b) factual information; or c) unrelated information (control). To ensure that participants continued to engage fully with the material, children were asked whether they would prefer to read the material aloud to the experimenter, or for the experimenter to read it to them. Next, children in each information group were randomly assigned to one of three counterbalancing groups and watched the automated vicarious learning procedure on the computer screen. Following this, children completed the second FBQ and then the NRT.

All children were fully debriefed at the end of the study and the aims were explained in age-appropriate language. Children were given the opportunity to ask any questions about the experiment and all questions were answered by the researcher. Factual information about the animals was given to the children both verbally and in a printed leaflet.

### Results

In all experiments an alpha level of .05 was used. Age was initially included as a covariate but was removed from the final analyses because it was not found to influence children’s responses significantly. Where there were nonsignificant results for the interaction effects that tested the core substantive hypotheses, Bayes Factors were estimated. Rather than asking whether or not effects are significant, Bayes Factors (BF_10_) quantify the ratio of the probability of the data under the alternative hypothesis relative to the null. A value of 1 means that the observed data are equally probable under the null and alternative hypotheses; values above 1 suggest that the data are more probable under the alternative hypotheses relative to the null; and values below 1 suggest that the data are more probable under the null hypotheses relative to the alternative. For example, BF > 3 suggests the data are three times more probable under the alternative hypothesis than the null, and the reciprocal (BF < 1/3) suggests the data are three times more probable under the null hypothesis than the alternative. Bayes Factors provide information about the probability of the data under the null hypothesis (relative to the alternative), whereas significance tests provide no evidence at all about the status of the null hypothesis (Dienes, 2014).

Bayes Factors were estimated using the *anovaBF* functions in the BayesFactor (Morey & Rouder, 2014) package in R (R Core Team, 2014). This function uses a default Jeffries prior (Rouder, Morey, Speckman, & Province, 2012). This default prior models prior beliefs in the effect size using a Cauchy distribution centered on 0 and with a default scale factor of 0.707. In doing so, our prior belief is that there is a 50% probability that the effect size (*d*) lies between −0.707 to 0.707. This default value represents a fairly open-minded belief that effects could range from fairly large and positive in the predicted direction to equally large in the opposite direction.

#### Fear beliefs

**Figure 1 fig1:**
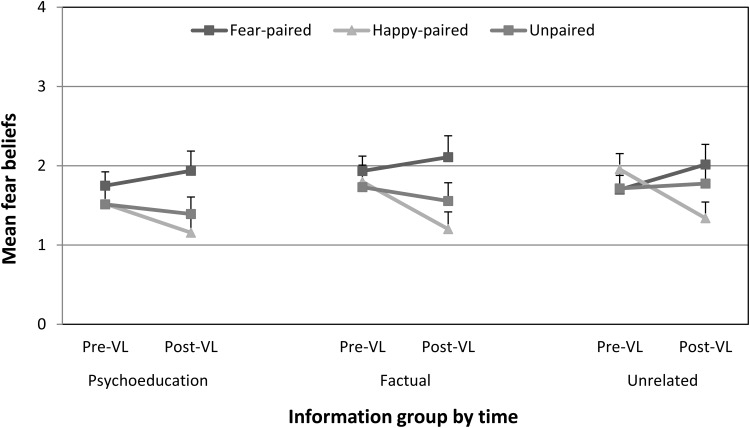
Mean (and *SE*) fear beliefs for fear-paired, happy-paired, and unpaired animals before and after vicarious learning (VL) in each of the three information groups in Experiment 1.

Figure 1 shows mean fear beliefs before and after vicarious learning in each of the three groups. A 3(group: psychoeducation, factual information, unrelated information) × 3(pairing type: fear-paired, happy-paired, unpaired) × 2(time: before vs. after vicarious learning) mixed ANOVA was performed on fear beliefs scores. There was no significant effect of group, *F*(2, 53) = 0.48, *p* = .62, η_p_^2^ = .02 (95% CIs [0, 0.11]), time × group interaction, *F*(2, 53) = 0.41, *p* = .67, η_p_^2^ = .02 (95% CIs [0, 0.10]), or pairing type × group interaction, *F*(4, 106) = 0.52, *p* = .72, η_p_^2^ = .02 (95% CIs [0, 0.06]). However, there was a significant main effect of pairing type, *F*(2, 106) = 7.46, *p* = .001, η_p_^2^ = .12 (95% CIs [0.02, 0.21]), and time, *F*(1, 53) = 5.10, *p* = .028, η_p_^2^ = .09 (95% CIs [0, 0.25]). More important, a significant (Greenhouse-Geisser) time × pairing type interaction indicated a significant change in fear beliefs over time depending on the type of pairing type children saw, *F*(1.68, 89.1) = 16.07, *p* < .001, η_p_^2^ = .23 (95% CIs [0.089, 0.36]). Follow-up planned comparisons found that fear beliefs had significantly increased overall for fear-paired animals, *F*(1, 53) = 7.44, *p* = .009, η_p_^2^ = .12 (95% CIs [0.0083, 0.29]), and significantly decreased for happy-paired animals, *F*(1, 53) = 12.86, *p* = .001, η_p_^2^ = .20 (95% CIs [0.039, 0.37]), compared to unpaired animals. However, the time × pairing type × group interaction, *F*(4, 106) = 0.57, *p* = .69, η_p_^2^ = .02 (95% CIs [0, 0.062]), BF_01_ = 0.059 (± 0.63%), was not significant and the effect size was small, showing that these changes in fear beliefs due to vicarious learning were statistically equivalent in all groups and were not significantly affected by the type of information children had received before learning. The Bayes Factor for this interaction compares the three-way interaction to the time × pairing type interaction, and therefore quantifies the evidence that the information group moderated this two-way interaction. The Bayes Factor indicates that the data are 16.95 times more probable under the null hypothesis than the alternative, which is overwhelming evidence that the information condition did not moderate the time × pairing type interaction.[Fig-anchor fig1]

#### Avoidance preferences

**Figure 2 fig2:**
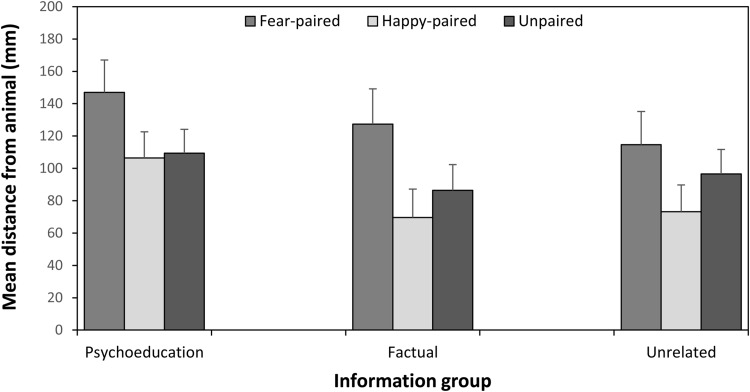
Mean (and *SE*) distances (avoidance preferences) from fear-paired, happy-paired, and unpaired animals in each of the three information groups in Experiment 1.

Figure 2 shows mean avoidance preferences for animals by pairing and group. A 3(group: psychoeducation, factual information, unrelated information) × 3(pairing type: fear-paired, happy-paired, unpaired) mixed ANOVA was performed on NRT distances. There was a significant (Greenhouse-Geisser adjusted) main effect of pairing type, *F*(1.62, 86.06) = 10.64, *p* < .001, η_p_^2^ = .17 (95% CIs [0.042, 0.30]). Planned comparisons revealed that in accordance with expectations, children’s avoidance preferences for fear-paired animals (*M* = 130.05, *SD* = 89.05) were greater than for unpaired animals (*M* = 98.07, *SD* = 65.15), *F*(1, 53) = 7.75, *p* = .007, η_p_^2^ = .13 (95% CIs [0.01, 0.30]). The difference between avoidance preferences for happy-paired (*M* = 83.95, *SD* = 73.18) and unpaired animals approached, but did not quite reach, conventional levels of significance, *F*(1, 53) = 3.74, *p* = .058, η_p_^2^ = .07 (95% CIs [0, 0.22]). The main effect of group, *F*(2, 53) = 1.14, *p* = .33, η_p_^2^ = .04 (95% CIs [0, 0.16]), and the pairing type × group interaction, *F*(3.25, 86.06) = 0.37, *p* = .79, η_p_^2^ = .01 (95% CIs [0, 0.056]), BF_01_ = 0.057 (± 0.41%), were not significant and effect sizes were small. This shows that children’s elevated avoidance preferences for fear-paired animals were similar in all groups and not significantly affected by the type of information they had received. The Bayes Factor for this interaction compares it to the main effect of pairing type, and therefore quantifies the evidence that the prevention condition moderated the effect of pairing type. The Bayes Factor indicates that the data are 17.54 times more probable under the null hypothesis than the alternative, which is overwhelming evidence that the information condition did not moderate the effect of pairing type.[Fig-anchor fig2]

## Experiment 2

Experiment 1 found that attempts to manipulate children’s beliefs about the US or CS using information did not inhibit fear-related learning during subsequent vicarious learning. One reason for this may be that the information and fear learning pathways were not matched: information was via written text, while fear learning was observational via pictures of emotional faces. Experiment 2 investigated the effectiveness of observational interventions to change children’s expectancies and prevent fear learning. One well-established example of the effect of CS-US expectancy evaluations on learning is “latent inhibition” (Lubow, 1989; Siddle, Remington, & Churchill, 1985): Conditioning models show that frequent exposure to a CS alone makes it more difficult for associations between the CS and a US to form during a later learning event. Latent inhibition effects occur because animals learn that there is no relationship between the CS and US, so the ability of the CS to predict the US is reduced (Field, 2006). For example, if children have had frequent nontraumatic experience with dogs it is less likely that they will learn to fear them in a later negative learning event involving a dog.

Related to latent inhibition is a concept that Mineka and Cook (1986) referred to as *immunization*. In a series of highly influential experiments, Mineka, Cook and colleagues demonstrated that non-snake-fearful lab-reared monkeys can learn to fear snakes after watching other monkeys acting fearfully with snakes (e.g., Mineka, Davidson, Cook, & Keir, 1984). In one of these experiments, six out of eight monkeys that had previously watched nonfearful models with snakes did not show fear-acquisition during observational learning, while a group of rhesus monkeys who had been given prior exposure to snakes (latent inhibition) did (Mineka & Cook, 1986). This suggests that immunization in the form of positive US modeling with the CS is a more potent method for inhibiting vicarious fear learning than neutral (CS alone) exposure in monkeys. Mineka and Cook speculated, though, that lack of latent inhibition could also have been the result of low sample size, and hence this result was less conclusive.

In a related study, Egliston and Rapee (2007) initially presented three groups of toddlers (12–20 months) with three conditions: positive maternal modeling with a stimulus (immunization); a stimulus-alone no-modeling condition (latent inhibition); or a no-stimulus or modeling condition (control). In support of Mineka and Cook’s findings, positive modeling but not stimulus-alone presentations inhibited the acquisition of fear responses during subsequent negative modeling with a fear-relevant stimulus (toy snake or spider) by the experimenter. Thus this study suggests that toddlers’ expectancies about the CS–US relationship can be changed, and fear learning inhibited, when the prevention pathway matches the vicarious learning pathway; that is, when both are observational learning. However, immunization in this study was modeled by the child’s mother, while subsequent observational fear learning was modeled by the experimenter. Thus one explanation for this finding could simply be that maternal modeling is more potent than modeling by strangers in toddlers. If the positive model had been a stranger, immunization may not have prevented learning, which would limit its practical use as a prevention strategy. In support of this, there is evidence that infants faced with novel ambiguous situations and stimuli use information from their mothers’, but not strangers’, responses (Zarbatany & Lamb, 1985).

Dunne and Askew have argued that school-age children are also accustomed to learning from nonfamily members and recognize that strangers can impart useful information about safety and danger in the environment. They demonstrated that when 6–10 year olds are given positive modeling following fear vicarious learning there is no significant difference between the effectiveness of maternal and stranger models, either in the strength of the initial vicariously learnt fear response, or subsequent counterconditioning: positive stranger models can reverse fear responses learnt from both strangers and mothers in this age group (Dunne & Askew, 2013). The current experiment will test whether Dunne and Askew’s predictions for this age group are also supported for prevention in immunization. In addition, Egliston and Rapee’s (2007) positive and negative models used facial expressions, vocal expressions, and physical gestures to express their emotions. One characteristic of Askew and Field’s (2007) paradigm is that it reduces the learning event to an elemental, mechanistic level of associations between a visual representation of the CS (animal) and US (model’s response), and has shown that just observing the model’s facial expression is sufficient for children to learn fear-related responses. In terms of theory and developing interventions it would be useful to know whether facial expressions are sufficient for immunization to occur. In addition, it would clarify the exact mechanisms underlying the effectiveness of positive modeling as a feature of some prevention programs with children.

In Experiment 2, children (7–10 years) were again divided into three groups prior to vicarious fear learning. Similar to Mineka and Cook (1986) and Egliston and Rapee (2007), this time one group received immunization in the form of positive modeling, one group received latent inhibition in the form of stimulus-alone presentations, and one group was a control. In addition to the older sample of children, another difference to previous studies was that, as in Experiment 1, a measure of children’s fear-related cognitions for animals was taken as well as a behavioral measure. It was expected that immunization would successfully inhibit learning in this age group. Although conditioning models predict that latent inhibition should prevent fear learning, this was not found in the context of vicarious learning with monkeys or toddlers. The researchers reported some ambiguity, however, in the evidence for monkeys, and findings for toddlers might be age-related. Given differences in cognitive ability between 7 and 10 year olds and toddlers, it was anticipated that latent inhibition might be more in line with the wider conditioning literature.

### Method

#### Participants

There were 105 children from three primary schools in Essex, U.K. (58 males and 47 females), with 35 children being randomly allocated from all three schools to each of the three groups. Children’s overall age range was 7.03 to 10.87 years with a mean age of 8.31 years (*SD* = 1.15). Mean ages in the three groups were no different (latent inhibition: *M* = 8.43 years, *SD* = 0.93 years; immunization: *M* = 8.35 years, *SD* = 0.98; no prevention: *M* = 8.40 years, *SD* = 0.76), *F*(2, 102) = 0.05, *p* = .95. As in Experiment 1, child-specific sociodemographic information was not collected but school records showed that children were predominantly White British and fluent English speakers at all three schools. Sixty children were from schools where a higher than average proportion of children were socioeconomically disadvantaged. Forty-five were attending a school with a lower than average proportion of socioeconomically disadvantaged children.

#### Materials

##### Stimuli

###### Animals (CSs)

Animal CSs consisted of 20 color pictures of two of the Australian marsupials (10 pictures of a quokka and 10 of a cuscus). Each picture measured 346 × 444 pixels.

###### Faces (USs)

Face USs were the 10 pictures of scared faces (five males and five females) and 10 pictures of happy faces (five males and five females) taken from the NimStim Face Stimulus Set (Tottenham et al., 2009). Each measured approximately 346 × 444 pixels.

##### Measures

###### FBQ

A 14-item computerized version of the FBQ was used in Experiment 2 for the quokka and cuscus: seven identical questions for each of the two animals. Internal consistency was acceptable before vicarious learning: Cronbach’s alpha = .64 (Cuscus subscale); .68 (Quokka subscale), and high after vicarious learning: α = .81 and .83, respectively.

###### NRT

Avoidance preferences for each animal was measured using the NRT. In Experiment 2, a green felt-covered rectangular board (680 mm × 500 mm) with pipe cleaner trees and fences was used as the nature reserve. Children were again asked to place a figure on the board where they would like to be and the distance between the figure and each animal was measured to determine approach or avoidance preferences for each child.

##### Preventative learning manipulation

###### Immunization

Children in the immunization group were presented with positive modeling for the animal they would later see in vicarious fear learning trials. Immunization consisted of 10 happy-pairing trials in which the fear-paired animal was seen together with happy faces. Trial timings were the same as for happy vicarious learning trials in Experiment 1. The unpaired animal was not seen during these trials.

###### Latent inhibition

During latent inhibition, children in this group saw unpaired presentations of the animal they would later see in fear-paired vicarious learning trials. There were 10 trials of the animal alone and timings were the same as for unpaired animals during vicarious learning in Experiment 1. The unpaired animal was not seen at all at this stage.

###### Control

Children in the control condition saw no prevention trials.

##### Vicarious learning

Vicarious learning was identical to Experiment 1 except that there were only two animals presented with scared or no faces. All timings were the same as for Experiment 1.

#### Procedure

The FBQs, preventative learning and vicarious fear learning were automated on a program written in E-Prime 2.0 by the second author on a Samsung RF511 Laptop. Children first completed the NRT followed immediately by the first (prelearning) FBQ. Next they were randomly allocated to one of the three groups and received either immunization, latent inhibition, or no prevention control trials. Following this, all children saw a series of randomized vicarious learning trials that were identical to Experiment 1 except that only two animals were used, so that during vicarious learning children saw one of the animals with scared faces and one with no faces. Because some of the children would already see one of the animals with happy faces in the prevention phase of the experiment, there was no happy-pairing condition during vicarious learning. Finally, the FBQ and nature reserve task were completed for a second time to explore changes in fear beliefs and avoidance preferences due to vicarious learning.

### Results

#### Fear beliefs

**Figure 3 fig3:**
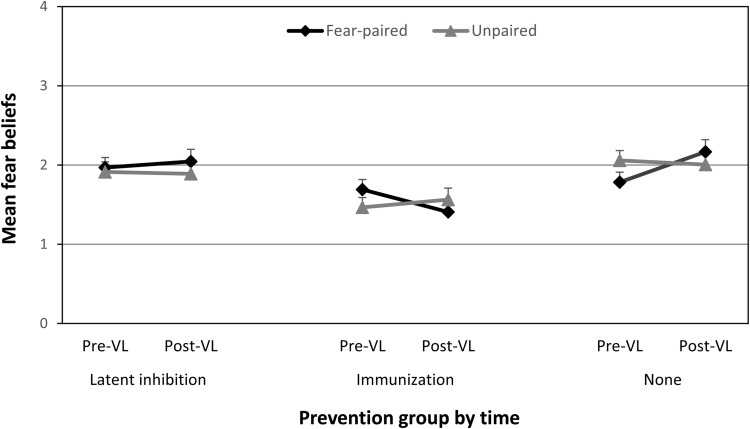
Mean (and *SE*) fear beliefs for fear-paired and unpaired animals before and after vicarious learning (VL) in each of the three information groups in Experiment 2.

Mean changes in fear beliefs for children in the latent inhibition group, immunization group and control group are displayed in Figure 3. A three-way 3(group: latent inhibition, immunization, control) × 2(pairing type: fear-paired vs. unpaired) × 2(time: before vs. after vicarious learning) mixed ANOVA was conducted on fear belief scores. There was a significant main effect of group, *F*(2, 102) = 5.99, *p* = .003, η_p_^2^ = .11 (95% CIs [0.013, 0.21]), but all other main effect and interactions were nonsignificant, except for the critical significant group × pairing type × time interaction, *F*(2, 102) = 5.24, *p* = .007, η_p_^2^ = .09 (95% CIs [0.008, 0.20]), BF_01_ = 2.60 (± 0.58%). The Bayes Factor indicates that when the three-way interaction is compared to the pairing type × time interaction, the data are 2.60 times more probable under the alternative hypothesis than the null. In other words, it shows modest evidence for the hypothesis that the prevention group moderated the effect of vicarious learning (as shown by the pairing type × time interaction). The significant three-way interaction was followed up with simple effects analyses comparing changes in fear beliefs (previcarious learning fear beliefs subtracted from postvicarious learning fear beliefs) for fear-paired and unpaired animals in each group. Results indicated that there was an expected significant increase in fear beliefs for fear-paired animals compared to unpaired animals in the control group, *F*(1, 102) = 5.92, *p* = .017, η_p_^2^ = .055 (95% CIs [0.002, 0.16]), BF_01_ = 1.34 (± 1.32%). However, there was no significant change in fear beliefs for fear-paired animals compared to unpaired animals in the latent inhibition group and the effect size was extremely small, *F*(1, 102) = 0.32, *p* = .57, η_p_^2^ = .003 (95% CIs [0, 0.057]), BF_01_ = 0.27 (± 1.67%). There was a significant decrease in fear beliefs for the fear-paired animals compared to unpaired animals in the immunization group, *F*(1, 102) = 4.50, *p* = .036, η_p_^2^ = .042 (95% CIs [0, 0.14]), BF_01_ = 1.60 (± 1.01%). These results suggest that latent inhibition prevented fear-learning, and immunization actually led to lower fear beliefs than at the outset of the experiment, despite vicarious fear-learning. The Bayes Factors confirm these conclusions although the evidence for increases in fear beliefs due to vicarious learning in the control group and decreases in fear beliefs due to immunization followed by vicarious fear learning was weak (the data were 1.34 and 1.6 times more probable under the alternative hypothesis compared to the null), the evidence for the absence of learning in the latent inhibition group was fairly strong (the data were three times more probable under the null than the alternative hypothesis).[Fig-anchor fig3]

#### Avoidance preferences

**Figure 4 fig4:**
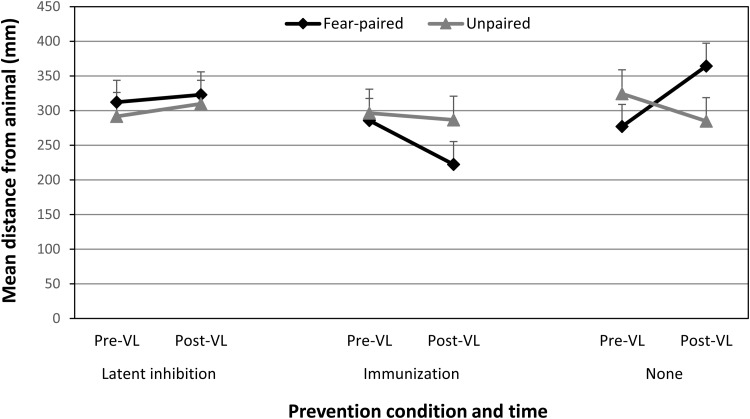
Mean (and *SE*) distances (avoidance preferences) from fear-paired, happy-paired, and unpaired animals in each of the three information groups in Experiment 2.

Figure 4 shows children’s avoidance preferences for animals in the latent inhibition, immunization and no prevention (control) groups. A three-way 3(group: latent inhibition, immunization, control) × 2(pairing type: fear-paired vs. unpaired) × 2(time: before vs. after vicarious learning) mixed ANOVA conducted on changes in avoidance preferences found a significant prevention group × time interaction, *F*(2, 102) = 3.84, *p* = .025, η_p_^2^ = .07 (95% CIs [0.0001, 0.17]). All other main effects and interactions were nonsignificant except for the significant group × pairing type × time interaction, *F*(2, 102) = 9.79, *p* < .001, η_p_^2^ = .16 (95% CIs [0.044, 0.28]), BF_01_ = 25.42 (± 0.63%). The Bayes Factor indicates that when the three-way interaction is compared to the pairing type × time interaction, the data are 25.42 times more probable under the alternative hypothesis than the null. This is very strong evidence for the hypothesis that prevention group moderated the effect of vicarious learning. This effect is important because it shows that the effect of vicarious learning on avoidance preferences was different in each group. The interaction was followed up with simple effects analyses comparing changes in avoidance preferences for fear-paired and unpaired animals in each group. In the control group, there was a significant increase in avoidance for fear-paired animals compared to unpaired animals, *F*(1, 102) = 17.93, *p* < .001, η_p_^2^ = .15 (95% CIs [0.044, 0.27]), BF_01_ = 19.59 (± 0.97%). In contrast, there was no significant change in avoidance preferences for fear-paired animals compared to control animals in the latent inhibition group and the effect size was extremely small, *F*(1, 102) = 0.06, *p* = .81, η_p_^2^ = .0006 (95% CIs [0, 0.24]), BF_01_ = 0.25 (± 1.86%); and in the immunization group avoidance preferences for fear-paired animals compared to unpaired animals actually approached significance decrease, *F*(1, 102) = 3.20, *p* = .077, η_p_^2^ = .03 (95% CIs [0, 0.12]), BF_01_ = 0.78 (± 1.27%). Thus, children in the latent inhibition and immunization groups did not show the significant vicariously learnt increases in avoidance preferences exhibited by children who did not receive a prevention procedure. The Bayes factors confirm these conclusions with very strong evidence for vicarious learning in the control group, strong evidence for the null in the latent inhibition group and fairly even evidence (slightly favoring the null) for no effect of vicarious learning or it having an effect (i.e., decreased avoidance) in the immunization group.[Fig-anchor fig4]

## Discussion

Two experiments investigated the inhibition of children’s vicariously learned fear responses for novel stimuli via information. Results found: (a) confirmation in Experiments 1 and 2 that fear vicarious learning leads to increases in children’s fear-related cognitions (fear beliefs) and behavior (avoidance preferences) for novel stimuli; (b) no evidence in Experiment 1 that written information interventions in the form of psychoeducation or factual information inhibit fear-related vicarious learning; and (c) evidence in Experiment 2 that increasing children’s visual familiarity for stimuli (latent inhibition), or providing positive observational learning (immunization), prevents children from vicariously learning fear-related cognitions and behavior.

### Theoretical Implications

The results support findings from multiple studies showing that vicarious learning increases children’s fear responses for novel animals (e.g., Askew, Cakir, Põldsam, & G. Reynolds, 2014; Askew et al., 2013; Askew & Field, 2007; Dubi et al., 2008; Gerull & Rapee, 2002; G. Reynolds et al., 2014; G. Reynolds, Field, & Askew, 2015a). The central aim of the studies was to investigate whether reducing threat expectancies for CSs would, as predicted by contemporary conditioning models, inhibit vicarious fear learning. Experiment 2 showed that positive modeling can “immunize” children against future vicarious fear learning, confirming predictions that vicarious fear learning shares this characteristic of conditioning models. It is well-established in the conditioning literature that prior learning history with a stimulus is a critical influence on whether fear develops during a negative learning event involving the stimulus (Field, 2006) and this was shown here to also be the case for vicarious fear learning in children. This result with 7–10 year olds builds on previous evidence from monkeys (Mineka & Cook, 1986) and toddlers immunized by mother models (Egliston & Rapee, 2007), unequivocally demonstrating that children in this age group are also influenced by the positive emotional responses of adult strangers. In fact, postlearning fear-related responses in the immunization group were actually significantly lower than at baseline here. Thus the findings contribute to our understanding of the etiology of fears and phobias, explaining how different individuals can experience the same traumatic vicarious learning event during childhood but only some will go on to develop fear.

Experiment 2 also demonstrated that prelearning neutral familiarity with a stimulus (latent inhibition) inhibits subsequent vicarious fear learning. Again, this establishes that vicarious learning shares this characteristic predicted by contemporary conditioning models, supporting the proposal that vicarious and conditioning share the same underlying mechanisms and processes. This contrasts with evidence from toddlers (Egliston & Rapee, 2007) and monkeys (Mineka & Cook, 1986), which indicated significant immunization but not latent inhibition. The reasons for this are not clear although one possibility is that latent inhibition requires more advanced cognitive skills than immunization and these skills are more developed in 7–10 year olds than toddlers or monkeys. For example, latent inhibition may require memory abilities not yet adequately developed in toddlers. However, how such abilities would differ from those needed for immunization is not clear as both would presumably require memory that a particular stimulus predicts a specific positive or neutral outcome. Also, previous evidence with monkeys was not entirely unequivocal because although there was no significant effect of latent inhibition on fear learning, monkeys in this condition did show less fear acquisition than controls, and Mineka and Cook speculated that with a larger sample this difference might have reached significance.

While latent inhibition prevented vicarious fear learning in Experiment 2, stimulus familiarity via nonthreatening information in Experiment 1 did not prevent vicarious learning. Inhibition of learning was only found then when both the preventative intervention and subsequent fear learning were delivered via the observational learning pathway. This supports a conclusion that modality of inhibition and learning is crucial: Inhibition is more potent when the pathway of delivery matches the fear-learning pathway. This echoes Kelly et al.’s (2010) finding that fear reduction was greater when the fear reversal pathway matched the acquisition pathway, except that fear acquisition and subsequent reversal were both via the information pathway in their study.

One potential limitation of the methodology was that there were no measures of expectancies and consequently it is not possible to distinguish between two possible explanations for the nonsignificant effect of information in Experiment 1: nonsignificance could be due either to the failure of information to change expectancies or a failure of changes in expectancies to prevent vicarious learning. In terms of the first explanation, there is substantial evidence that positive verbal information can decrease children’s fear beliefs for novel animals (e.g., Field, Argyris, & Knowles, 2001; Field & Lawson, 2003), and positive information about animals may be more effective at reducing threat expectancies for threatening outcomes than the relatively neutral, nonthreatening information used here. However, past evidence suggests that even overtly positive information may not be sufficient to inhibit subsequent vicarious fear learning (Askew, Kessock-Philip, & Field, 2008). Thus the second explanation may be more likely: that information did successfully reduce threat expectancies in Experiment 1, but did not successfully inhibit vicarious learning because of the mismatch between the modality of learnt expectancies and fear learning. As discussed, this is in contrast to Experiment 2 where both successful prevention and fear learning were delivered via the observational pathway.

Attempts to devalue children’s interpretation of models’ responses using psychoeducation did not prevent vicarious fear acquisition in Experiment 1. Devaluation of US (the models’ responses) intensity would be expected to reduce the magnitude of a CR formed during learning. Children’s attitudes to USs were not directly tested in the current procedure; therefore, it is not possible to ascertain whether the failure of psychoeducation was because it did not sufficiently alter children’s interpretations of models’ fear responses (i.e., beliefs about the negativity of the US), or because altered beliefs about the US did not inhibit learning because they were not in the same modality. In the first scenario, vicarious learning may simply have been more effective than the information transmitted earlier via psychoeducation. Ten models’ faces were seen during vicarious learning and children may have reasoned that if so many individuals were afraid of the animal, evidence that the animals were threatening outweighed the information that sometimes individuals can fear stimuli that are not actually dangerous. To test this, future research should test psychoeducation as a preventative manipulation prior to vicarious learning involving a single model. Another possibility is that psychoeducation would more effectively inhibit vicarious learning if presented in the same modality as it. Future research could test this; for example, by having psychoeducation presented in films, or verbally by the experimenter or another model. It is also worth noting that there was no measure of how engaged children were with the information they were given and it may be that more detailed one-to-one discussion with children would be needed to maximize engagement sufficiently for the information to effectively inhibit vicarious learning.

### Clinical Implications

The findings have implications for parents, teachers, clinicians, and others working with children because they confirm that nonthreatening familiarity with a stimulus can protect children from learning to fear the stimulus from others. Positive modeling was particularly effective in this regard, and actually resulted in decreased fear beliefs for the stimulus following fear-related vicarious learning. Given that specific phobias typically begin during childhood (Öst, 1987; Öst & Treffers, 2001) and often persist for many years before the individual seeks treatment (e.g., Wang et al., 2005), successful prevention and early intervention strategies during childhood have the potential to alleviate many years of suffering. The current study confirms that positive modeling has an effective role in prevention programs and that associative learning underpins this effect. The results suggest that immunization may be a more useful method of preventing fear development in 7 to 10-year-olds than early counterconditioning because it does not rely on the presence of a positive model after the vicarious learning event.

It is possible that immunization generalizes to other sets of similar stimuli and this should be investigated in future research. However, one disadvantage of immunization as a general prevention strategy is that it may require specific stimuli to be identified and targeted. To ensure maximum effect, immunization might most usefully be targeted at common “fear-relevant” stimuli for which fears are most likely to develop in childhood (e.g., spiders and snakes). Research should confirm that immunization and latent inhibition are also effective at preventing vicarious fear learning for these stimuli in this age group when delivered by stranger models, as it is in toddlers (Egliston & Rapee, 2007). Psychoeducation was not effective here. However, because it targets children’s cognitions for models’ responses (USs) to stimuli, and not a specific stimulus (CS), it could potentially be used to prevent vicariously learnt fears more widely than immunization if an effective intervention were developed.

Given that positive modeling occurred directly before negative learning in the current procedure, it is still unclear how enduring this protection is, and future research should examine whether the effectiveness of immunization diminishes when the time interval before vicarious learning increases. The results suggest that how the learning history is acquired is critical: prevention appears to be more successful when the modality of the learning history matches the modality of the current learning event. However, modality was not manipulated in a single experiment, and future research that directly compares prevention modalities would be necessary to determine unequivocally whether prevention is superior when there is a match.

By 7 to 10 years, children are likely to already be familiar with many animals and so future research should look at whether similar effects are also found (a) for stimuli that children in this age group are already familiar with, including common phobic stimuli such as snakes; and (b) for younger children who are likely to have had less experience of stimuli in general and are therefore potentially even more vulnerable to fear learning. Clinically, it would also be useful to investigate whether these contemporary conditioning properties also apply to children particularly at risk of anxiety disorders, such the offspring of mothers with anxiety. Finally, the research investigated fear learning for novel animals, but other types of fears, such as social anxiety, can also be vicariously learnt (Askew, Hagel, & Morgan, 2015). Future research should investigate whether these fears can also be prevented using immunization and latent inhibition.
